# Protocol Design, Compliance, and Feasibility of Frequent Remote Monitoring in Patients Receiving Lecanemab

**DOI:** 10.1002/alz70858_106208

**Published:** 2025-12-26

**Authors:** Alexa Goldstein, Erin Anita Krahn, Neguine Rezaii, Mark C. Eldaief, Scott M McGinnis, Raseeka O’Chander, Bonnie Wong, Brad C. Dickerson

**Affiliations:** ^1^ Frontotemporal Disorders Unit, Massachusetts General Hospital, Boston, MA, USA; ^2^ Frontotemporal Disorders Unit, Department of Neurology, Massachusetts General Hospital and Harvard Medical School, Boston, MA, USA; ^3^ Frontotemporal Disorders Unit and Massachusetts Alzheimer's Disease Research Center, Department of Neurology, Massachusetts General Hospital and Harvard Medical School, Boston, MA, USA; ^4^ Frontotemporal Disorders Unit and Massachusetts Alzheimer's Disease Research Center, Department of Neurology, Massachusetts General Hospital and Harvard Medical School, Boston, MA, USA; ^5^ Brigham and Women's Hospital, Boston, MA, USA; ^6^ Department of Psychiatry, Massachusetts General Hospital, Boston, MA, USA

## Abstract

**Background:**

Anti‐amyloid disease‐modifying therapies (DMTs) for Alzheimer's Disease (AD) such as lecanemab are being increasingly prescribed. This mandates more frequent and in‐depth tracking of patients receiving these agents in order to better estimate their cognitive, behavioral, and functional trajectories while receiving treatment. Here we report the feasibility of a frequent cognitive testing protocol by telehealth for AD patients receiving lecanemab.

**Method:**

18 AD patients receiving lecanemab as part of their clinical care were enrolled in a prospective observational study. Participants completed validated questionnaires assessing cognition, mood, behavior, and functional status (Amsterdam IADL; NPIQ) in REDCap prior to their baseline telehealth cognitive assessment. Following their baseline session, telehealth assessments were performed every 3 months, and included a modified AD Assessment Scale‐Cognitive (ADAS‐Cog) (with alternate forms at each timepoint, in keeping with the procedures often used in AD clinical trials). Baseline and monthly testing sessions included additional tests of memory, language, and executive function with alternate forms through the platform SurveyLex. For each telehealth session, participants viewed task stimuli over Zoom with the examiner present; responses were audio recorded by the SurveyLex software.

**Result:**

34 potential participants have been screened thus far. 24% declined participation due to the demands of testing and timing. 53% have enrolled (55% female; average age=67.2 ± 9.5 years). Participants included a range of AD syndromes (MCI/mild‐dementia, PCA, PPA). Average MoCA score of those enrolled (obtained prior to beginning infusions) was 19.2 ± 4.92. Baseline and 3‐month sessions lasted an average of 67.07 minutes (range: 45‐120 minutes), while shorter monthly sessions by SurveyLex lasted an average of 34.93 minutes (range: 24‐45 minutes). Five participants withdrew due to the demands of testing; mean MoCA score for these participants was 20.8 ± 5.26 (range: 14‐27). Enrolled participants have completed an average of 3.6 monthly visits thus far (range: 1‐9).

**Conclusion:**

Preliminary results indicate that frequent remote cognitive testing of AD patients receiving lecanemab is feasible. Compliance appears to be moderate with 61% of participants remaining enrolled 3 months after their first visit. We propose remote monthly assessments as a new model to monitor patients receiving DMTs for AD.

